# Right-Sided Cardiac Hydatid Cyst Complicated With Pulmonary Embolization: A Pediatric Case Report

**DOI:** 10.7759/cureus.11503

**Published:** 2020-11-16

**Authors:** Maryem Assamti, Hammam Rasras, Nabila Ismaili, Noha Elouafi

**Affiliations:** 1 Cardiology, Mohammed VI University Hospital, Mohammed First University, Oujda, MAR

**Keywords:** cardiac echinoccocosis, pulmonary embolism, cardiac imaging, surgery

## Abstract

Echinococcosis is an endemic zoonotic parasitic infection found in Morocco, due to *Echinococcus granulosus*. Cardiac localization is a rare form, presenting in various ways. The right heart is an uncommon site of cardiac involvement. We report a case of a right-sided cardiac hydatid cyst complicated with multiple pulmonary embolism, revealed by dyspnea at rest, in a 15-year-old male. The patient successfully underwent surgical removal which confirmed the diagnosis. This case report demonstrates one of the various clinical presentations of this condition, and the importance of early treatment in order to prevent its potentially fatal complications, particularly in the young.

## Introduction

Echinococcosis is a parasitic infection that represents a major public health problem in endemic areas. It usually involves the liver or lungs. The cardiac involvement is reported in only 0.02%-2% of cases [1]. The revealing symptoms vary from chest pain to angina-like pain, shortness of breath, palpitations, cough, and hemoptysis, or unexplained fever [2]. Thus, cardiac echinococcosis requires a high index of suspicion in endemic areas, such as in Morocco. The differential diagnosis includes intracardiac tumors, parietal thrombi, or abscess. Given the possible life-threatening complications, early surgical removal should be considered even in asymptomatic patients [3].

## Case presentation

We report a case of a 15-year-old male, from a rural area, complaining of shortness of breath, dry cough, and fever, since three weeks before his admission. He was treated with antibiotics, and there was no improvement. Initial echocardiography revealed an intracardiac mass associated with a mobile element suspended to the tricuspid valve. A tricuspid valve endocarditis was initially suspected, and the patient was referred to our hospital with this preliminary diagnosis.

During the physical examination, the patient was conscious and cooperative. His vitals and respiratory sounds were normal. The patient’s liver and spleen were nonpalpable, without any dermatologic lesions. The electrocardiogram showed sinus rhythm with a heart rate of 50 bpm.

**Figure 1:**
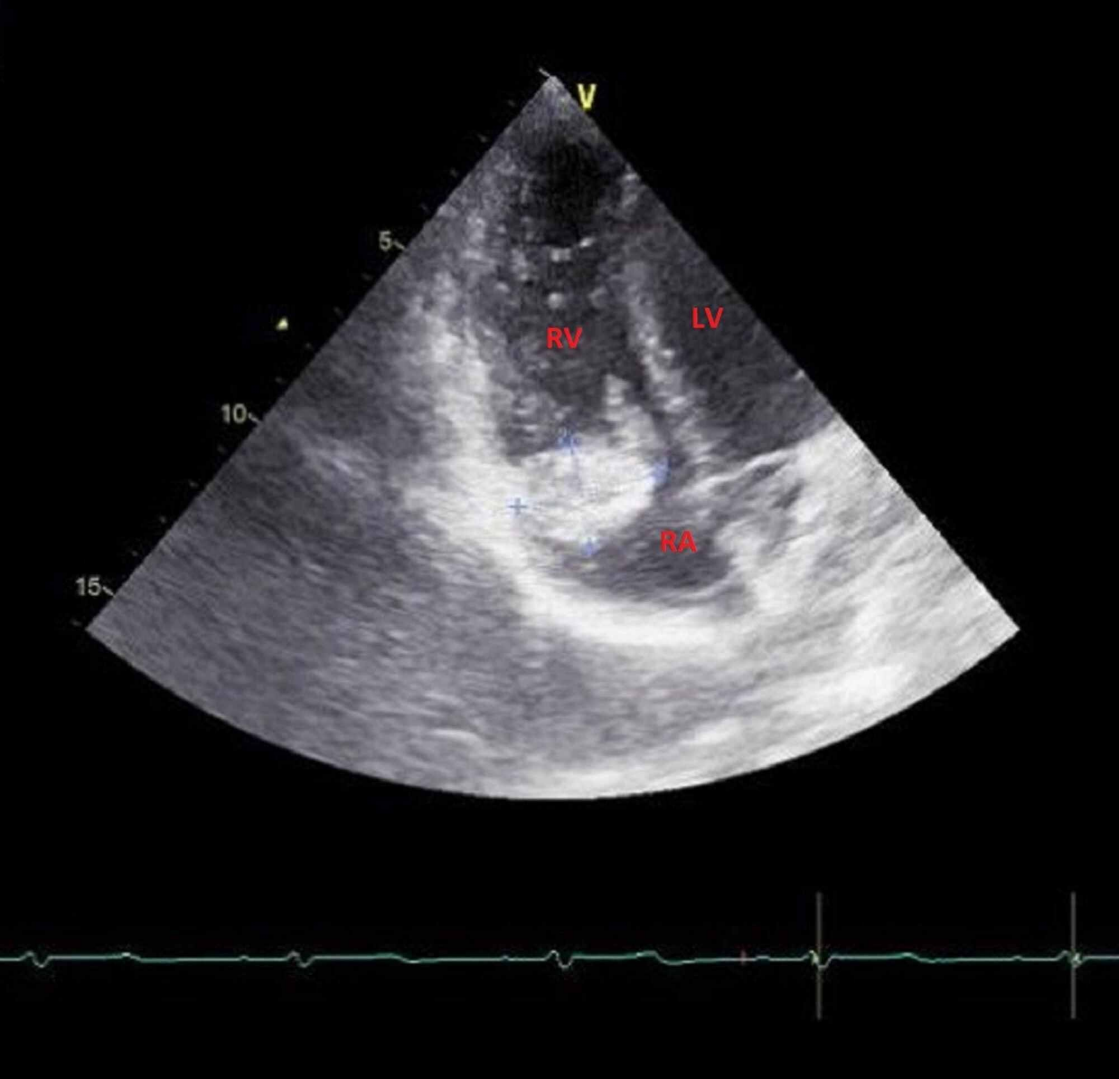
Four-chamber view showing a hyperechogenic heterogeneous mass in the RA with a mobile element suspended to the anterior tricuspid leaflet. RV, right ventricle; RA, right atrium; LV, left ventricle

**Figure 2:**
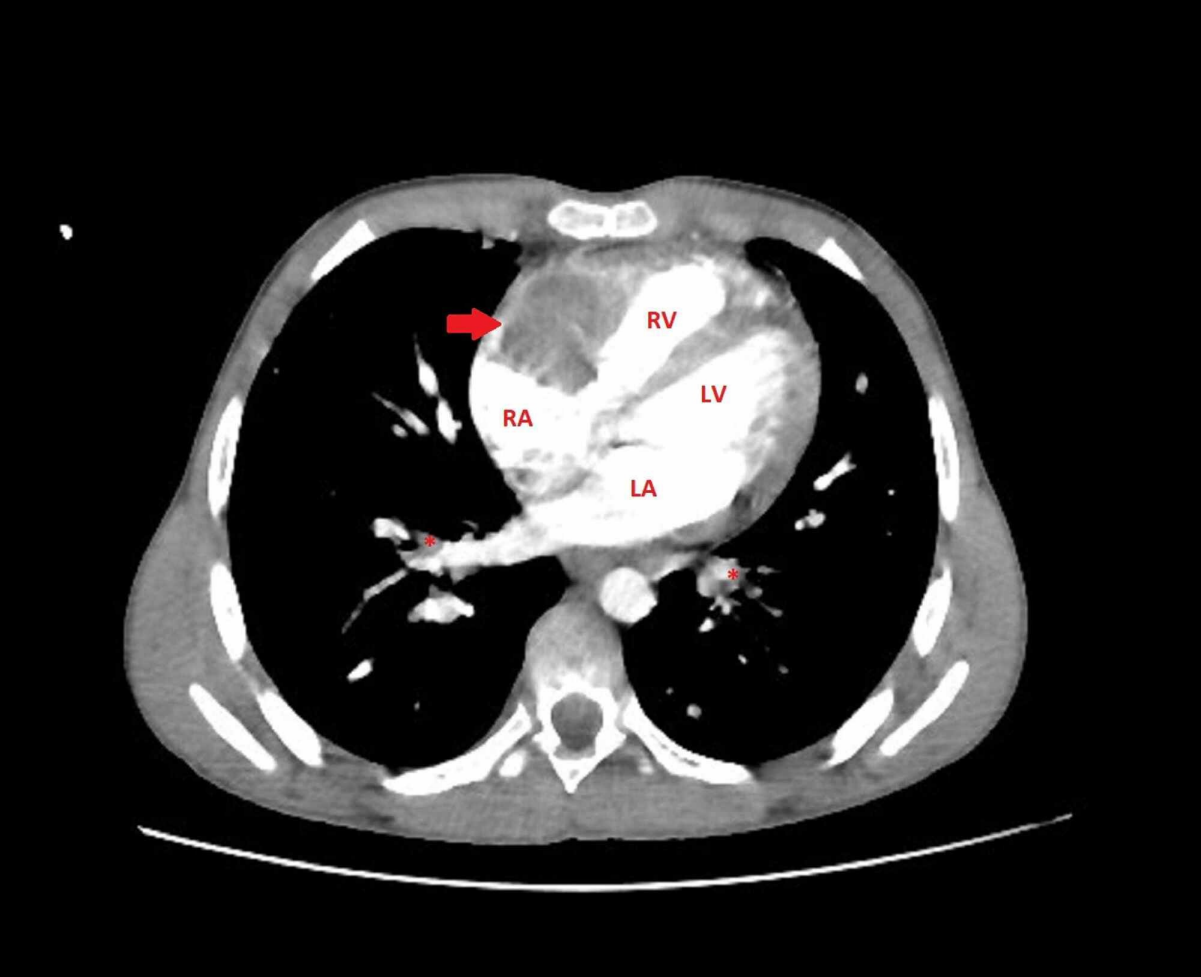
Chest CT scan with contrast showing a well-defined hypodense mass (red arrow) of 35 mm x 32 mm, partially calcified that straddles between the RA and the RV, associated with similar pulmonary lesions located in the segmentary branches of the left inferior lobar artery marked with a red asterisk. RV, right ventricle; RA, right atrium; LV, left ventricle

**Figure 3:**
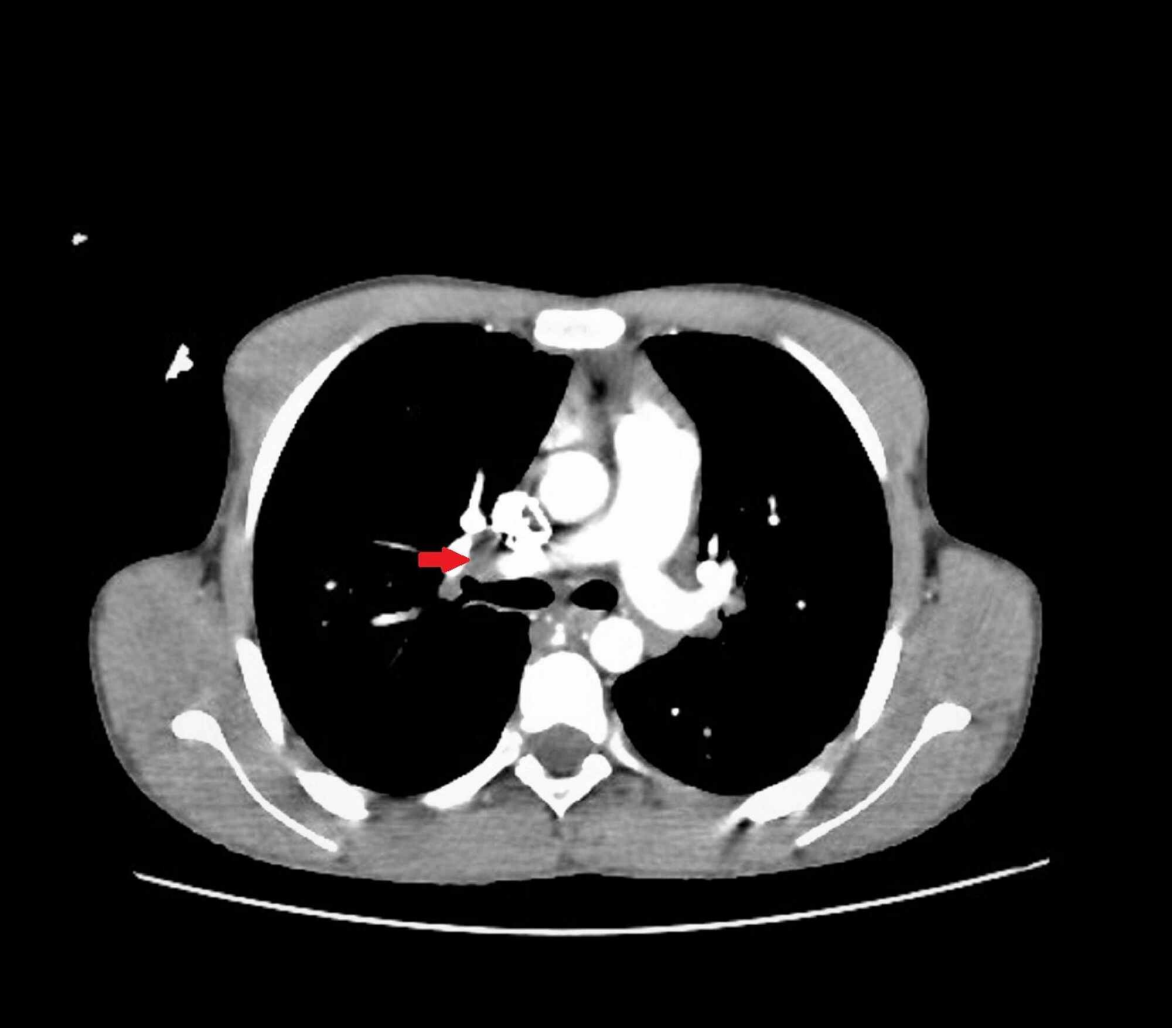
Chest CT scan showing a lobar inferior pulmonary embolism (red arrow).

Echocardiography showed a well-defined hyperechogenic heterogeneous intracardiac mass in the right atrium, of 32 mm x 34 mm, with a mobile element suspended to the anterior tricuspid leaflet (Figure 1, Videos 1-3), associated with pulmonary hypertension. The patient underwent a thoracic CT scan that showed a well-defined hypodense mass of 35 mm x 32 mm, partially calcified that straddles between the right atrium and the right ventricle, associated with similar pulmonary lesions located in the right inferior lobar artery and segmentary branches of the left inferior lobar artery, with multiple nodular parenchymatous lesions (Figures 2-3). A cranial and abdominal CT scan revealed no additional lesions.

**Video 1:** Modified parasternal long-axis view on transthoracic echocardiography.

**Video 2:** Apical four-chamber view on transthoracic echocardiography.

**Video 3:** Apical five-chamber view on transthoracic echocardiography.

A complete blood count showed a hypereosinophilia (at 10%), with an accelerated erythrocyte sedimentation rate of 65 mm/hour and a negative C-reactive protein. A set of three blood cultures remained sterile. An indirect hemagglutination test for hydatid disease was positive (with a titer of 1:1280).

The patient was transferred to the cardiothoracic surgery department. Anthelmintic therapy was initiated, and the patient underwent a cyst excision of the intracardiac mass with a De Vega tricuspid annuloplasty, on cardiopulmonary bypass. The macroscopic findings confirmed the diagnosis of hydatid cyst. Pulmonary emboli were not accessible to excision, the patient was treated with albendazole 15 mg/kg/day and was discharged after one week. No incident occurred during a three month follow-up. The patient remained asymptomatic (Video 4).

**Video 4:** Transaortic parasternal short-axis view of the follow-up echocardiography.

## Discussion

Cardiac hydatid cysts were described for the first time by Williams in 1836 [4]. This infectious disease is primarily carried by dogs and cats. Humans can be an aberrant intermediate host if they ingest the parasite eggs. A cardiac hydatid cyst is usually the primary lesion related to hematogenous or lymphatic dissemination [5].

Hydatid cyst affects the left heart rather than the right heart, probably due to its large mass and major blood supply. The most common location is the left ventricular free wall (55%-60%), followed by the right ventricle (15%) and the interventricular septum (5%-9%). The right atrium is involved in only 3%-4% of cases [3].

Only 10% of cardiac hydatid cyst patients are symptomatic [6]. The disease remains silent for years, due to the slow increase in cyst diameter (about one to 1.5 cm per year) [7]. Depending on its location, the enlargement of the cyst may lead to the right or left ventricular outlet obstruction, valvular insufficiency or stenosis, cardiac failure, compression of coronary arteries, arrhythmia, or atrioventricular and intraventricular blocks [4].

The rupture often reveals intracardiac cysts and could lead to systemic embolization of the germinative membrane or a secondary cyst (pulmonary, cerebral, or coronary ...), cardiac tamponade, or anaphylaxis increasing the mortality [3]. Hydatid pulmonary embolism is a rare complication that may be due to intracardiac cyst rupture (such as in our case), or rarely due to dissemination from extracardiac locations. It may be lethal or evolve to chronic pulmonary hypertension [8].

The diagnostic approach for cardiac hydatidosis is based on imaging and serological test. Enzyme-linked immunosorbent assay is the most specific and sensitive technique [1].

Transthoracic echocardiography remains a key screening tool that demonstrates the cyst, its location and limits, and its hemodynamic impact. Nevertheless, the differential diagnosis of a ruptured cyst is challenging; it includes myxoma, rhabdomyoma, lipoma, teratoma, or a thrombus. CT and MRI may then provide valuable data. Pathognomonic signs include the presence of daughter cysts, peripheral calcifications, and membrane detachment [4]. The anatomical mapping is crucial to guide surgical removal.

Surgical treatment is the modality of choice in the management of cardiac hydatid cysts, and it should be considered even in asymptomatic patients. The surgical approach depends on the location of the cyst. Medical antiparasitic therapy before surgery must be avoided, as this may cause cyst death and thinning of the cyst wall and eventually its rupture [3].

Post-operative medical therapy decreases recurrence significantly and should, therefore, be applied [9]. The recommended germicide is 10-15 mg/kg/day of albendazole for six months [10]. Others suggest exclusive medical therapy in case of small or calcified cysts or inoperable cases [11].

## Conclusions

Cardiac hydatid cyst is a rare yet critical variant of the parasitic infection. We reported this case to underline its atypical clinical and radiological presentations. Despite the rarity of this disease, its complications may be fatal. Whatever the location, surgical removal of the cyst is the definitive treatment, in addition to medical therapy.
